# Analysis of Regional Financial Risk in Guangdong Province Based on the DCN Deep Learning Model

**DOI:** 10.1155/2022/9274737

**Published:** 2022-07-20

**Authors:** Yan Yuan

**Affiliations:** Guangdong Mechanical & Electrical Polytechnic, School of Economics and Trade, Guangzhou 510515, China

## Abstract

In the free flow of financial factors oriented to capital, returns will be accompanied by the concentration and diffusion of financial resources to form regional financial spatial differences, which is an objective phenomenon of regional financial practice. Localized regional financial risks may appear in the process of regional financial practice in each region. To address the abovementioned problems, we propose a model for regional financial risk analysis based on the DCN deep learning model. The main contents are as follows: elaborating the financial risk transmission mechanism involving intra- and interregional financial risks, sorting out the relationship between sectors as clues; the designing process of regional financial risk index as well as the measurement method, and the regional financial risk index for typical regions is measured and found to be at peak in 2017 with a risk index of 0.58; and the construction of an early warning model based on the value of the regional financial risk index and the expansion of the RNN network applied to the construction of the regional financial risk early warning system. Based on the construction of the RNN network application risk early warning system, the three types of risks, payment risk, loan loss risk, and market risk with the percentages of 49.62%, 26.82%, and 23.56%, respectively, are derived, and the focus is on their supervision and management in the follow-up work.

## 1. Introduction

**Figure 1:**
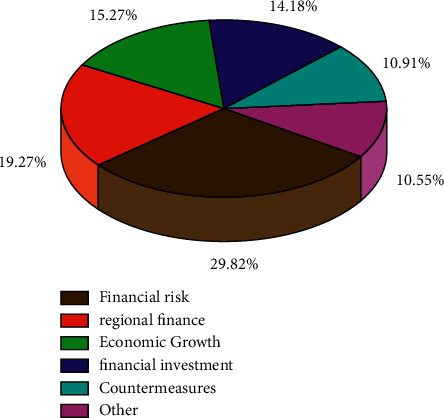
Regional economic research topics proportion chart.

The cyclical movements and adjustment fluctuations of the global economy in all major regions can be seen as a combination of financial risk phases erupting and subsiding in time [1–3]. The international financial crisis that erupted in 2008 was a major cross-regional financial risk event in recent years, which plunged the world economy into the longest, most extensive and deepest downward adjustment since the Second World War. Different from the previous adjustment, the path of economic stagnation, and natural liquidation towards recovery, after this adjustment, each major economy successively relied on the power of government policy, using extremely loose macro policy to implement a major bottoming out for the briefly stagnant economy. At present, China's reform and opening up is developing in depth, and the regional economy is also in the stage of continuous development. At the same time, regional economic operation is an indispensable part of the national economic development process, is an intermediate level between macroeconomics and microeconomics, is to ensure the stable development of the regional economy and the sustainable development of the national economy as a whole, and will play an important role of stability and coordination. Regional economic development in the sustained development of the national economy is to have an important role: first of all, there is innovation and breakthrough role. Due to the multiple economic development models developed in each region [3, 4], which are the result of proactive economic development and strengthening of regional economic management, there is an important contribution to the economic development of the region and other regions. Second, it has a stabilizing and coordinating effect. On the one hand, because of the large differences in the resources and production capacities possessed by each region, regional economic management can promote the exchange of products and strengthen the economic ties between different regions, thus ensuring the economic stability of each region and realizing the complementary advantages of different regions. On the other hand, regional economic management can self-regulate faster and implement the mechanism of national macromanagement and regional hierarchical management, which makes the whole management system perform more flexible and effective. For the study of regional economic development, we analyzed the regional economic research theme direction through the database, as shown in Figure 1, in the regional economic research, and financial risk research is the most critical [1, 2, 5], accounting for 29.28%, followed by regional economy and economic growth and financial investment. In order to ensure stable growth of regional economy, considering financial risks in the context of actual situation and based on regional perspective, it is a necessary path for regional economic development.

The analysis of financial risks for regional economic development is gathered in the relationship between financial risks and regional characteristics which are the specific triggers of financial risks and the evolution of financial risks for regional economic development afterward. In recent years, there have been many regional financial risks in China, such as local private lending, usury, and other financial risks with insufficient control [3, 4] and lack of effective isolation, all of which can easily lead to the collapse of financial risks. Strategiesand characteristics of regional economic development in China's regional economic development strategy refers to the planning and decision-making on issues related to the overall, long-term and critical economic and social development in a certain region. To be more specific, it refers to the total planning and decision-making of the guiding ideology of regional economic development, the goals to be achieved, the priorities to be solved, and the stages to be experienced as well as the countermeasures that must be taken in a longer period of time, based on the estimation of the regional economic and social development, taking into account all aspects of the regional economic [6–9] and social development. Financial risk is mainly due to the uncertainty of financial activities causing losses of participants' subjects, which are latent accumulation and suddenly dramatic and have a strong contagious diffusion. Feng and Yuan [10] studied the interaction between the financial structure and financial risk, its multifractal asymmetric version and time delay based detrended intercorrelation, and market-oriented financial structure which increases financial risk; moreover, the financial structure and financial risk and their interaction is characterized by multifractal, asymmetric, and bidirectional transmission. Song et al. [11] proposed an early warning method for corporate financial risk. The results show that the random forest algorithm can more objectively and intelligently obtain the basic probability assignment of evidence theory. Gonzalez and Quast [12] studied the relationship between sovereign risk and financial risk, and a large part of the linkage between sovereign spreads is caused by changes in global financial risk. Regional financial risk issues are related to the economic development of the region, diverse and complex, and at the same time contagious. In addition, regional financial risk is related to regional finance, and the current state of financial development in different regions makes the regional tolerance for financial risk different. Due to the inconsistency of regional economies, financial risk indices are not at all consistent. Therefore, regional financial risk is also the focus of research efforts. Huang et al. [13] performed correlation analysis from regional, financial, and global stock indices, and in their research work, they proposed a model for measuring systematic risk based on dynamic topological indicators and introduced a topological parametric model to measure systematic risk and compare it with traditional measurement models. The results show that the new model can provide more detailed and accurate information about the systematic risk of the stock market. The method can be used for investment decision recommendation and systematic risk warning. Tao [14] set the explanatory variables of financial stress index; through the analysis of the research of various scholars, we found that the deep learning model algorithm plays a vital role in the analysis of regional economic and financial risk.

Regional financial risks are distinctive because of the characteristics of regional economic development. For regional financial risks, common inducing factors include payment risk due to illiquidity, loan loss risk, and market risk. Guangdong Province is the leading province in China's regional economic development. Since 1989, Guangdong has been ranked first in GDP in China for 33 consecutive years, and the economy of Guangdong Province is pivotal in the national economy. The economic structure and regional economic characteristics of Guangdong Province are like a smaller version of China. Although Guangdong has a huge volume, the economic growth rate is still stable at 6.3% at the medium to high speed level. Therefore, this paper analyzes the financial risks related to the regional economy of Guangdong Province, hoping to contribute to the rapid and stable growth of the regional economy of Guangdong Province. This paper analyzes and predicts the financial risks related to the regional economy of Guangdong Province by considering three main factors: payment risks, loan loss risks, and market risks related to the regional economy through deep learning algorithms.

## 2. Regional Financial Risk-Related Concepts

Financial liquidity is subject to regional limitations [15–17], and there are large objective differences between different regional economic environments and financial systems. This inter-regional economic and geographical limitation and inter-regional differences in factors make financial risks occur in a horizontal spatial perspective with a regional basis and can exhibit regional characteristics. Regional financial risk is an integral risk in the region and contains regional and integral risk characteristics. Regional financial risk is a dynamic and continuous change, which is a brittle process of accumulation and development in the region. The characteristics of the main influencing factors for regional financial risk are shown in Table 1.

For the generation of regional financial risk, it mainly stems from two main reasons: the fragility and instability of finance itself and the distortion of the real environment in the region. The fragility of finance is a state shown by the gradual loss of its own ability to resist risks in the process of operation and is an important reason for the generation of financial risks and financial instability. The micro perspective, or financial vulnerability in the narrow sense [18, 19], refers to the nature of financial institutions that are more prone to failure and that failure will have significant consequences due to the industry characteristics of operating with leveraged liabilities. The macro perspective, that is, financial vulnerability in the broad sense, refers to a financial state in which the financial system often tends to be highly risky in its self-operation and in its daily interactions with the outside world. For financial vulnerability, it is often caused by information asymmetry, homogeneity of market participants, too much homogeneity in the process of interaction between the government's real sector for macro policies, too much improper government intervention, the distorted reality of the regional passing environment, serious local government debt problems, high leverage of real enterprises resulting in unstable regional economic development, structural imbalance, imbalance of local industrial structure, local economic development relying solely on a single industry, insufficient development of new high-tech multifaceted industries, overcapacity in traditional industries, etc.

### 2.1. Feasibility of Recurrent Neural Networks for Financial Risk Analysis

Recurrent neural network (RNN) is developed based on the neural network and introduces directional loops, that is, the input of the hidden layer at each moment, thus reflecting the backward and forward sequence relationship between the input data. The regional economy is a microcosm of the national economy and is comprehensive and regional in nature. In recent years, convolutional neural networks such as residual networks and dense structured networks have been proposed one after another continuously refreshing the accuracy of classification tasks on data sets, and the recognition ability of some categories even surpasses that of humans. Therefore, the development of convolutional neural networks has driven the rapid development of computerized financial risk analysis, which has become a mainstream method.

### 2.2. Structure of Recurrent Neural Network

In this paper, a recurrent neural network (RNN) is used for speech recognition. Recurrent networks can process sequences of arbitrary lengths to prevent the computational results from overfitting or underfitting the dataset [20, 21]. The network calculation error can be reduced by dropout, regularization, and orthogonalization. The following is a detailed introduction to its operation:

(1) Dropout. It refers to randomly freezing a portion of the neurons in the hidden layer from the fully connected neural network in each iteration of training by reducing the correlation between neurons and the complexity of the model in such a way that the effect of regularization can be achieved
(2) Regularization. It reduces the test set error at the cost of increasing the training set error to improve the generalization ability of the model. Currently, the three main regularization methods include parametric penalty, dropout, and early stopping
  (a) Parametric penalty. By limiting the number of nonzero parameters, the *L*_0_ parametric number (the number of nonzero elements in the vector) is added to the cost function as a penalty term. The specific calculation results are as follows:
    (1) $$\begin{array}{c} \bar{L}\left(w,b\right)=L\left(w,b\right)+\frac{\lambda }{2m}\Omega \left(w\right), \end{array}$$
  where *L*(*w*, *b*) is the function before parametric penalty, *m* refers to the number of samples, *λ* represents a hyperparameter to control the degree of regularization, and Ω(*w*) denotes the regularization term
  The *L*_1_ parametrization is the optimal convex approximation of the *L*_0_ parametrization, and the minimization problem of the *L*_0_ parametrization can be transformed into the minimization problem of the *L*_1_ parametrization under certain conditions. The specific calculation results are as follows:
    (2) $$\begin{array}{c} \bar{L}\left(w,b\right)=L\left(w,b\right)+\frac{\lambda }{2m}{\left\|w\right\|}_{1}, \end{array}$$
  where *w*_1_ is the sum of the absolute values of the elements in the *L*_1_ parametrization vector
  *L*_2_ regularization is the change of the cost function penalty term from the sum of the absolute values of the elements of the vector in *L*_1_ regularization to the square of each element squared sum of the elements, that is, the square of the mode of the vector. The specific calculation results are as follows:
    (3) $$\begin{array}{c} \bar{L}\left(w,b\right)=L\left(w,b\right)+\frac{\lambda }{2m}{\left\|w\right\|}_{2}^{2}, \end{array}$$
  where *w*_2_ is the square root of the sum of the squares of the elements of the vector
  (b) Dropout. Randomly freezes a portion of the implicit layer neurons from the fully connected neural network at each iteration of training, reducing the correlation between neurons and the complexity of the model in such a way that the effect of regularization is achieved
  (c) Early stopping. For a specific early stop rule, a hyperparameter patience is usually set to stop training when the test error does not decrease in consecutive patience iterations

### 2.3. Learning Algorithms for  RNN Networks

RNNs are a class of recurrent neural networks and are recursive in the evolutionary direction of the sequence. In recurrent neural networks, the weight parameters *W*, *U,* and *V* are shared at each time step, thus greatly reducing the number of parameters to be learned in the network. The training of recurrent neural networks is the process of continuously correcting the weight parameters *W*, *U,* and *V*, using a back-propagation algorithm over time. The specific computational procedure is as follows:

(4) $$\begin{array}{c} s_{t}=f\left(Ux_{t}+Ws_{t-1}\right), \end{array}$$

where *s*_*t*_ is the state of the implied layer at time *t* and *x*_*t*_ is the input at moment *t*.

(5) $$\begin{array}{c} o_{t}=f\left(Vs_{t}\right), \end{array}$$

where *o*_*t*_ is activation functions, including sigmoid, tanh, and softmax functions. The specific calculation process is as follows:

(6) $$\begin{array}{c} \text{Sigmoid forward propagation }f\left(z\right)=\frac{1}{1+e^{-z}},\\ \text{Sigmoid back propagation }f{\left(z\right)}^{\prime }=f\left(z\right)\left(1-f\left(z\right)\right). \end{array}$$

The sigmoid function maps the input value to the interval between [0, 1], and the function is more sensitive to the signal change in the central area and less sensitive to the signal change in the two sides.

(7) $$\begin{array}{c} \text{tanh forward propagation }f\left(z\right)=\frac{e^{z}-e^{-z}}{e^{z}+e^{-z}},\\ \text{tanh back propagation }f{\left(z\right)}^{\prime }=1-f{\left(z\right)}^{2}. \end{array}$$

The tanh function can be seen as a variant of the sigmoid function, which maps the input value to the interval between [−1, 1]. The derivative of the function is generally larger than the sigmoid function, resulting in faster convergence during training.

(8) $$\begin{array}{c} \text{softmax forward propagation }f\;\left(z_{j}\right)=\frac{e^{z_{j}}}{\sum_{j}z_{j}},\\ \text{softmax back propagation }\frac{\delta \alpha_{j}}{\delta z_{j}}=\left\{\begin{array}{c} \alpha_{i}\left(1-\alpha_{j}\right),\\ -\alpha_{i}\alpha_{j}. \end{array}\right. \end{array}$$

Softmax function's output is mapped to the [0, 1] interval, and the sum of the inputs of all output layer neurons is 1. After applying the activation function, the output value of the output layer represents the relative probability of belonging to a certain category.

### 2.4. Deformable Convolution Module

**Figure 2:**
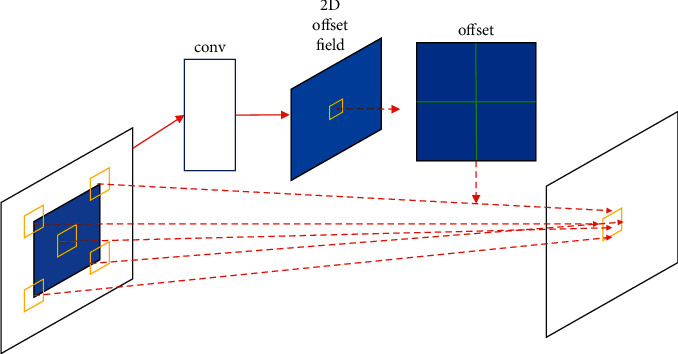
Variable convolutional architecture.

Therefore, this paper adds a deformable convolution to the output layer to extract more robust features, allowing the network to learn from the rectangular box to learn the data changes autonomously. Features are extracted from the data through different convolution kernels, and these features can be used to preserve the spatial relationship between the data, as shown in Figure 2.

It mainly includes two steps: sampling the input feature *X* using a specified step size S and a rectangular grid *R*; multiplying the data in the sampling area by the corresponding weight matrix, and then summing and outputting the corresponding data *y*, and the formula is as follows:

(9) $$\begin{array}{c} y\left(p_{0}\right)=\sum_{p_{0}\in R}w\left(p_{n}\right)\cdot x\left(p_{0}+p_{n}\right), \end{array}$$

where *p*_0_ is the output point on the output *y* and *p*_*n*_ is the set of positions on the convolution kernel *R*.

For the value of each position *p*_0_ on the output feature map *y*, the standard convolution is regularly sampled within the grid, while the deformable convolution adds an offset to the regular sampling:

(10) $$\begin{array}{c} y\left(p_{0}\right)=\sum_{p_{0}\in R}w\left(p_{n}\right)\cdot x\left(p_{0}+p_{n}+\Delta p_{n}\right), \end{array}$$

where Δ*p*_*n*_ is offset.

Add the offset Δ*p*_*n*_ to the original sampling position and then the new sampling position becomes *p*_*n*_ + Δ*p*_*n*_, but the offset is usually a floating-point number, so it needs to be solved by bilinear interpolation. The bilinear interpolation formula is as follows:

(11) $$\begin{array}{c} x\left(p\right)=\sum_{q}G\left(q,p\right)\cdot x\left(q\right), \end{array}$$

where *p* is *p*_0_ + *p*_*n*_ + Δ*p*_*n*_, *G*(*q*, *p*) is a two-dimensional bilinear interpolation kernel function. The two one-dimensional multiplication formulas are disassembled as follows:

(12) $$\begin{array}{c} G\left(q,p\right)=g\left(q_{x},p_{x}\right)\cdot g\left(q_{x},p_{x}\right). \end{array}$$

## 3. Experimental Verification and Comparative Analysis

For the experimental parameters, this paper trains the model by migration learning, and the backbone network uses a network pretrained on the dataset. The pretraining can stabilize the gradient change in the initial stage of the network and help to improve the accuracy rate. In this paper, the Adm descent method is used as the optimization algorithm of the model, the momentum value is set to 0.92 and the canonical factor is set to 0.0007, and the canonical term is set for overfitting in this paper. The base learning rate is 0.01, the batch size is set to 32, and the decay coefficient of the base learning rate is 0.1. When the network is close to the optimal solution, the learning rate needs to be reduced by the decay coefficient. Through the statistics of the number of training samples, the network trained according to different input data is more robust.

### 3.1. Empirical Analysis

The more common approach to financial risk measurement is the composite index model, which has the advantage of simplicity and clarity and can be used in combination with many complex models, expanding the scope of the method. The reality of the relatively late development of China's financial markets makes this study lack sufficient sample data and historical extreme scenarios to accurately estimate the model parameters.

This paper goes to determine whether the principal component analysis method is applied to determine the weight of each dimensional indicator. The test is more applied in factor analysis of multivariate statistics mainly by analyzing the sum of squared simple correlation coefficients; when the former is greater than the latter, the number is close to 1, which means that the correlation among variables is very strong and factor analysis can be performed. The KMO and Bartlett test results obtained in this study are shown in Table 2, which shows a KMO test value of 0.738 > 0.6. Meanwhile, the Bartlett spherical test value is 1078.215 and Sig is 0.000, indicating that the original hypothesis is rejected and the original variables can be correlated by principal component analysis, which can be used to construct a regional financial risk index model.

### 3.2. Model Optimization

On top of the traditional indicators, this paper then considers the search index risk measurement submodel constructed on the basis of RNN to reconstruct the index analysis. Four major regions are selected for this study to carry out the analysis of regional financial risk. The general situation is generally considered as a period of high stress when the degree of deviation is greater than twice its standard deviation, and the specific formula and determination criteria are as follows.

(13) $$\begin{array}{c} {\text{FSII}}_{it}=\frac{{\text{FSII}}_{it}\text{MEAN}\left(\text{FSI}\right)}{2\times \text{SD}\left({\text{FSI}}_{it}\right)}-1, \end{array}$$

where FSII_*it*_ is the regional financial risk stress identification index of the *i*-th region in period *t*, and FSI_*it*_ is the regional financial risk index in period *t*.

**Figure 3:**
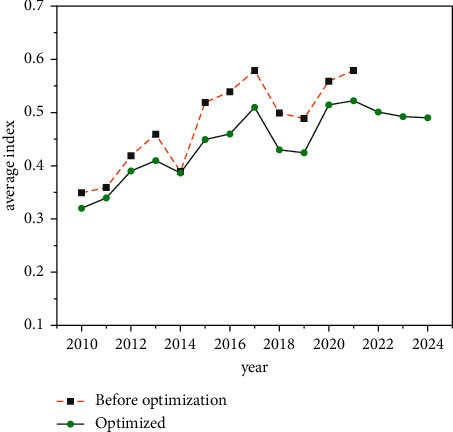
Evolution of the average regional financial risk index in Guangdong Province.

The study of this paper explores in detail the evolution of the actual regional financial risk index in Guangdong Province, which is elevated as shown in Figure 3, and finds that, in general, the regional financial risk in the country and in each region is on an upward trend compared with 2010 with different and fluctuating changes in the process. The average regional financial risk index of Guangdong Province peaked at 0.47, 0.58, and 0.57 in 2013, 2017, and 2021, respectively, indicating that there is still significant risk pressure on regional financial risk in China in recent years. By region, the regional financial risk in the central and western regions of Guangdong Province has been greater than that in the eastern and northeastern regions, requiring this paper to focus on prevention and mitigation. In summary, this is inextricably linked to the good economic and social conditions in these regions, the complete financial infrastructure and the strong local financial supervision as well as the ability to mitigate risks, and it is worthwhile to summarize the successful experience of all other regions in China.

**Figure 4:**
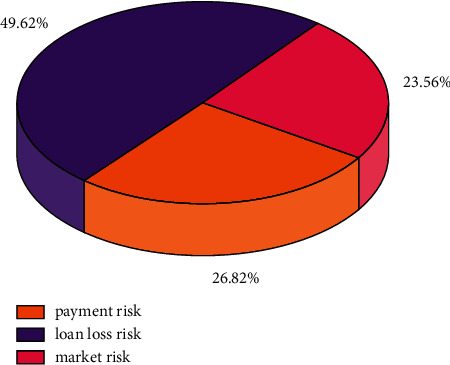
Evolution of the average regional financial risk index in Guangdong Province.

This paper compares the three types of risks, such as payment risk, loan loss risk, and market risk, through the model software as shown in Figure 4. It can be seen that the loan risk accounts for nearly half of the risk, which indicates that personal lending is very risky and has a great impact on the economic risk of Guangdong region, so we focus on its supervision and management in the follow-up work. In addition, payment risk and market risk account for 26.82% and 23.56%, respectively. They also need to be further optimized for research and analysis.

## 4. Conclusion

This paper systematically summarizes the existing comprehensive regional financial risk measurement system, including the construction methods of financial risk measurement and financial risk early warning system, in order to provide a theoretical reference system for subsequent research. This paper systematically summarizes the existing regional financial risk measurement system, including the construction methods of financial risk measurement and financial risk early warning system, in order to provide a theoretical reference system for subsequent studies.

1. We elaborate on the financial risk transmission mechanism involving intra and interregional financial risks and sort out the relationship between sectors as a clue to discover a complete and systematic regional financial risk transmission mechanism.
2. The regional financial risk index design process and the measurement method and the regional financial risk index measurement for typical regions fully exploit and use such data to facilitate the analysis of the real-time dynamic advantages of financial risk, making up for the shortcomings of traditional basic indicators.
3. The early warning model based on regional financial risk index values is constructed to extend the application of RNN networks to the construction of regional financial risk early warning system. It both extends the early warning system for building regional financial risks and provides an effective means for risk monitoring by relevant regulatory authorities.
4. Based on the construction of the RNN network application risk warning system, three types of risks, namely, payment risk, loan loss risk, and market risk, with the percentages of 49.62%, 26.82%, and 23.56%, respectively, were derived, which will be focused on the supervision and management in the follow-up work.

## Figures and Tables

**Table 1:** Characteristics of the nature of the main influencing factors of regional financial risk.

Characteristic features	Specific explanations
Negative externality	Regional financial risks may affect the geographical area several times and its origin and negative externalities may increase exponentially

Cryptogenic cumulative	Financial risks are the result of a long evolutionary build-up and it is difficult to predict when they will arrive

Complexity	Financial systems are increasingly complex and subject to multiple influences at the same time, making it difficult to anticipate risks

Multiple spaces depend on each other	Regional financial risks are interdependent with multiple spaces and there is a risk of spillover from the next external space

Multidimensional contagion	Regional financial risk correlation mechanisms are multidimensional and have broad contagion

**Table 2:** KMO and Bartlett test results table.

Kaiser-Meyer-Olkin metrics		0.738
Bartlett's sphericity test	Approximate chi-square	1078.215
	d*f*	98
	Sig.	0.000

## Data Availability

The dataset can be accessed upon request.

## References

[1] Zhu W., Zhang T., Wu Y., Li S., Li Z. (2022). Research on optimization of an enterprise financial risk early warning method based on the DS-RF model. *International Review of Financial Analysis*.

[2] Liu C., Fan Y., Xie Q., Wang C. (2022). Market-based versus bank-based financial structure in China: from the perspective of financial risk. *Structural Change and Economic Dynamics*.

[3] Danisewicz P., Schaeck C. H., Schaeck K. (2022). Private deposit insurance, deposit flows, bank lending, and moral hazard. *Journal of Financial Intermediation*.

[4] de Jong A., Veld T., Veld C. (2022). Legal risk and information spillover through private lender reports. *Journal of Financial Markets*.

[5] Boukhatem J. (2022). How does financial risk affect sukuk market development? empirical evidence from ARDL approach. *Heliyon*.

[6] Bai Y., Zhao M., Li R., Xin P. (2022). A new data mining method for time series in visual analysis of regional economy. *Information Processing & Management*.

[7] Zhou B., Wen Z., Yang Y. (2021). Agglomerating or dispersing? spatial effects of high-speed trains on regional tourism economies. *Tourism Management*.

[8] Zając P., Avdiushchenko A. (2020). The impact of converting waste into resources on the regional economy, evidence from Poland. *Ecological Modelling*.

[9] Chowdhury S. K., Endres M. L. (2021). The influence of regional economy- and industry-level environmental munificence on young firm growth. *Journal of Business Research*.

[10] Feng W., Yuan H. (2021). Haze pollution and economic fluctuations: an empirical analysis of Chinese cities. *Cleaner Environmental Systems*.

[11] Song M., Ma X., Shang Y., Zhao X. (2020). Influences of land resource assets on economic growth and fluctuation in China. *Resources Policy*.

[12] Gonzalez F., Quast T. (2022). The relationship between abortion rates and economic fluctuations. *Economics and Human Biology*.

[13] Huang G., Yao X., Yao X. (2015). Dynamics of China’s regional carbon emissions under gradient economic development mode. *Ecological Indicators*.

[14] Tao Z. (2011). Strategy of city development in low-carbon economic mode-a case study on qingdao. *Energy Procedia*.

[15] Zhang Y., Qian T., Tang W. (2022). Buildings-to-distribution-network integration considering power transformer loading capability and distribution network reconfiguration. *Energy*.

[16] Jia X., Zhang W., Zhang C. (2022). Commodity financialization and funding liquidity in China. *The North American Journal of Economics and Finance*.

[17] Zhou W., Zhong G. Y., Li J. C. (2022). Stability of financial market driven by information delay and liquidity in delay agent-based model. *Physica A: Statistical Mechanics and Its Applications*.

[18] Faiella I., Lavecchia L., Michelangeli V., Mistretta A. (2022). A climate stress test on the financial vulnerability of Italian households and firms. *Journal of Policy Modeling*.

[19] Qian T., Chen X., XinXin Y., Tang W., Wang L (2022). Resilient decentralized optimization of chance constrained electricity-gas systems over lossy communication networks. *Energy*.

[20] Qian T., Liu Y., Zhang W., Tang W., Shahidehpour M. (2020). Event-triggered updating method in centralized and distributed secondary controls for islanded microgrid restoration. *IEEE Transactions on Smart Grid*.

[21] Fang C., Tao Y., Wang J. (2021). Mapping relation of leakage currents of polluted insulators and discharge arc area. *Frontiers in Energy Research*.

